# Extraskeletal Ewing Sarcoma: Report of an Extremely Rare Case in Temporal Region

**Published:** 2018-11

**Authors:** Shahryar Bashiri, Hossein Heidar, Milad Parvin

**Affiliations:** 1 Postgraduate Student, Department of Oral and Maxillofacial Surgery, Shariati Hospital, Tehran University of Medical Sciences, Tehran, Iran; 2 Assistant Professor, Department of Oral and Maxillofacial Surgery, Sina Hospital, Tehran University of Medical Sciences, Tehran, Iran; 3 Postgraduate Student, Department of Oral and Maxillofacial Surgery, Shariati Hospital, Tehran University of Medical Sciences, Tehran, Iran

**Keywords:** Sarcoma, Ewing, Neoplasms, Temporal Muscle

## Abstract

Extraskeletal Ewing sarcoma (EES) is an uncommon tumor with low prevalence in the head and neck region. Herein, we report a 13-year-old boy with EES in the temporal region, which was managed by surgery and chemotherapy. The histological characteristics and the clinical manifestations of the lesion and our surgical approach will be discussed as well.

## INTRODUCTION

Ewing sarcoma (ES) is an invasive malignant tumor characterized by small round blue cells. It is often found in long bones and the pelvis during childhood [1]. Extraskeletal ES (EES) is an uncommon variant of ES, which more commonly occurs in the soft tissue of the lower extremities and the trunk rather than the bones. The paravertebral region is a prime location for EES [2,3].

Tumors that primarily occur in the head and neck region are often found in the nasal cavity, oral cavity, paranasal sinuses or the soft tissue of the neck [4,5]. Herein, we report a case of EES occurring in the temporal region of a 13-year-old boy.

## CASE REPORT

clinic of our institute complaining of a swelling in his right temporal region developed one month earlier. Clinical examination revealed a palpable swelling above the zygomatic arch and behind the lateral orbital rim, which became more prominent when the patient clenched his teeth.

**Fig. 1:**
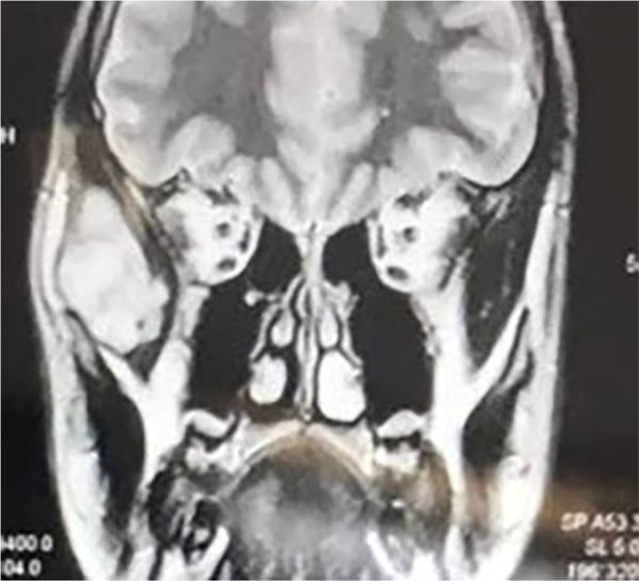
Magnetic resonance imaging (MRI) showing the lesion at the right temporal region

The patient had no pain, trismus or tenderness. Redness was not seen either. Neurological assessment was unremarkable. The patient did not report any weight loss. Magnetic resonance imaging (MRI) revealed a hyperintense well-defined mass in the right temporal fossa measuring 4 cm × 2.5 cm (Fig. 1).

An open biopsy was scheduled for the patient. We approached the lesion by making an incision over the temporal region and above the zygomatic arch. The lesion was located medial to the temporalis muscle. It contained a cystic fluid which was aspirated. The lesion did not have any adhesion to the surrounding tissues and was easily dissected.

**Fig. 2:**
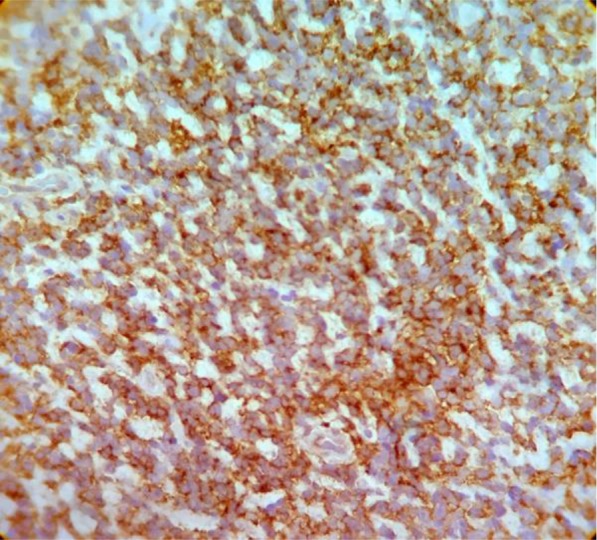
Immunohistochemical (IHC) staining for CD99 (100× magnification)

**Fig. 3:**
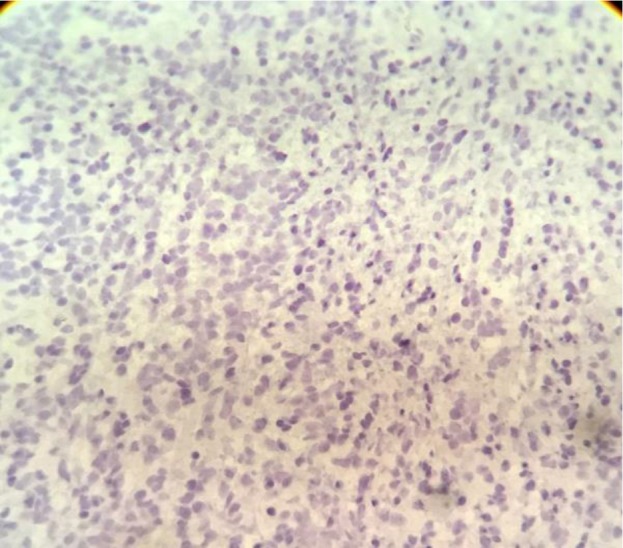
Immunohistochemical (IHC) staining for S100 (100× magnification)

**Fig. 4:**
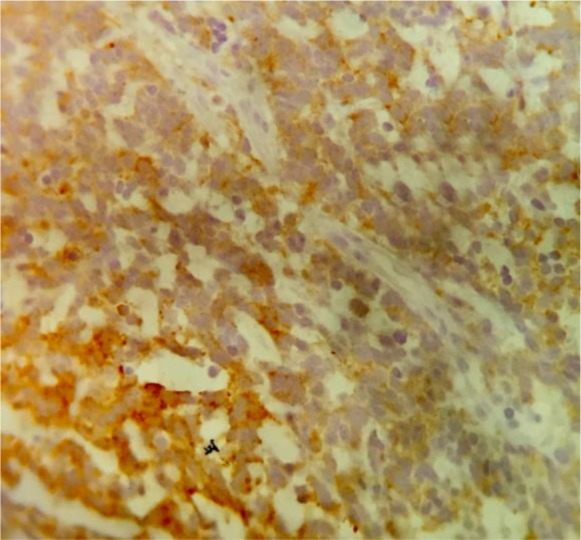
Immunohistochemical (IHC) staining for NSE (100× magnification)

Hematoxylin and eosin (H&E) staining revealed atypical cells with round to oval nuclei and prominent nucleoli. Immunohistochemical (IHC) staining revealed positive CD99 (Fig. 2), S100 (Fig. 3), and NSE (Fig. 4); accordingly, the diagnosis of EES was made.

The patient was referred to an oncology clinic where he underwent a whole body positron emission tomography (PET) scan which showed no metastasis or primary origin. The patient underwent 14 sessions of chemotherapy with doxorubicin, etoposide, ifosfamide, cyclophosphamide, mesna, actinomycin D, and vincristine. He has been followed up for 3 years so far with no evidence of recurrence or metastasis and has an excellent lifestyle.

## DISCUSSION

EES is a rare tumor accounting for 1.1% of soft tissue malignancies. It usually affects males between the ages of 15 and 30 years and has a high recurrence rate [6]. Its clinical and radiological features are nonspecific, and the diagnosis requires histopathological and IHC examinations. Regarding our patient, both clinical and radiological data suggested a dermoid cyst considering the involved region and the age of the patient [7]. In addition, the well-defined borders of the lesion on MRI images were highly suggestive of a cyst or benign tumor; therefore, an open biopsy was planned. After histopathological study and IHC staining, data revealed that tumor cells were positive for CD99, S100, and NSE; therefore, the diagnosis of EES was made.

Localized EES is treated by surgical excision in combination with adjuvant or neoadjuvant chemotherapy. In the present case, the surgical margins were free from the tumor, and the patient was referred to an oncology clinic and received 14 sessions of chemotherapy. Fortunately, he had good prognostic factors such as the absence of metastases, tumor size less than 10 cm in diameter, and clear surgical margins.

Due to fallacious characteristics of these lesions, one must care when facing such clinical and radiologic manifestations, especially when they present at unusual sites. Clinical features tend to be nonspecific and may mimic common benign conditions.

## CONCLUSION

Our presented case highlights the significance of the inclusion of EES in the list of differential diagnoses of facial soft tissue lesions. IHC staining is highly applicable for such cases. After reaching a diagnosis of EES, an invasive multimodal treatment is imperative.
